# Correlates of physical activity and sedentary behaviour in children attending before and after school care: a systematic review

**DOI:** 10.1186/s12889-022-14675-8

**Published:** 2022-12-16

**Authors:** Andrew J. Woods, Yasmine C. Probst, Jennifer Norman, Karen Wardle, Sarah T. Ryan, Linda Patel, Ruth K. Crowe, Anthony D. Okely

**Affiliations:** 1 School of Health and Society, Faculty of the Arts Social Sciences and Humanities, University of Wollongong, Wollongong, NSW Australia; 2 Early Start, Faculty of the Arts Social Sciences and Humanities, University of Wollongong, Wollongong, NSW Australia; 3 School of Medical Indigenous and Health Sciences, Faculty of Science Medicine and Health, University of Wollongong, Wollongong, NSW Australia; 4 Illawarra Health and Medical Research Institute, University of Wollongong, Wollongong, NSW Australia; 5 Health Promotion Service, Illawarra Shoalhaven Local Health District, Warrawong, NSW 2502 Australia; 6 Health Promotion Service, South Western Sydney Local Health District, Liverpool, NSW 2170 Australia

**Keywords:** Out of school hours care, After school program, Before school care, Physical activity, Sedentary behaviour, Review

## Abstract

**Background:**

Out of School Hours Care (OSHC) offers structured care to elementary/primary-aged children before and after school, and during school holidays. The promotion of physical activity in OSHC is important for childhood obesity prevention. The aim of this systematic review was to identify correlates of objectively measured physical activity and sedentary behaviour in before and after school care.

**Methods:**

A systematic search was conducted in Scopus, ERIC, MEDLINE (EBSCO), PsycINFO and Web of Science databases up to December 2021. Study inclusion criteria were: written in English; from a peer-reviewed journal; data from a centre-based before and/or after school care service; children with a mean age < 13 years; an objective measure of physical activity or sedentary behaviour; reported correlations and significance levels; and if an intervention study design these correlates were reported at baseline. Study quality was assessed using the Office of Health Assessment and Translation Risk of Bias Rating Tool for Human and Animal Studies. The PRISMA guidelines informed the reporting, and data were synthesised according to shared correlations and a social ecological framework.

**Results:**

Database searches identified 4559 papers, with 18 cross-sectional studies meeting the inclusion criteria.There were a total of 116 physical activity correlates and 64 sedentary behaviour correlates identified. The most frequently reported correlates of physical activity were child sex (males more active), staff engaging in physical activity, an absence of elimination games, and scheduling physical activity in daily programming (all more positively associated). The most frequently reported correlates of sedentary behaviour were child sex (females more sedentary) and age (older children more sedentary).

**Conclusions:**

Encouraging physical activity engagement of female children, promoting positive staff behaviours, removing elimination elements from games, and scheduling more time for physical activity should be priorities for service providers. Additional research is needed in before school care services.

**Supplementary Information:**

The online version contains supplementary material available at 10.1186/s12889-022-14675-8.

## Background

Childhood overweight and obesity is a critical public health issue [1]. Recent global estimates indicate that over 18% of children and adolescents aged 5–19 years have overweight or obesity, compared with just 4% in 1975 [2]. The World Health Organization [3] attributes the increased prevalence of childhood obesity to a global shift in diets towards energy-dense, nutrient-poor foods; and a trend towards less physical activity (PA) due to increasingly sedentary lifestyles. Managing childhood overweight and obesity will require population-based, multi-sectoral and multi-disciplinary approaches [3].

A setting which provides an opportunity for an environmental level approach to childhood overweight and obesity is Out of School Hours Care (OSHC). OSHC offers care to elementary/primary-aged children before and after school, and during school holidays, with an average of 29% of children aged 6 to 11 years across Organisation for Economic Co-operation and Development (OECD) countries attending centre-based before and/or after school care services [4].

While OSHC services can have a positive impact on the PA and healthy eating of children through active play and the provision of healthy snacks [5, 6], childhood obesity interventions in OSHC settings have been mixed and generally ineffective in reducing child obesity (e.g. body mass index (BMI), body composition, cardiovascular fitness) [7]. A review of obesity interventions in after school care services found many interventions were focussed on increasing PA but not on reducing sedentary activities [7]. Reducing sedentary behaviour is important given its association with several adverse health outcomes [8]. To the authors’ knowledge, no reviews have systematically looked at factors that are associated with child PA and sedentary behaviour while attending OSHC services. Understanding the influences on and thus, potential, causes of PA behaviours has been widely identified as important for evidence-based planning of public health interventions [9].

The aim of this systematic review was to identify correlates of objectively measured PA and sedentary behaviour in before and after school care. While vacation care, such as summer camps, is considered an OSHC service, this review included only before and after school care, given the differences in programming and delivery compared with vacation care. Consistent with other reviews of PA and sedentary behaviour in children [10–12] a social ecological framework was used in correlate categorisation to provide an organised multilevel approach to help inform future interventions in the OSHC setting [13].

## Methods

The reporting of this review followed the 2020 Preferred Reporting Items for Systematic Reviews and Meta-analysis (PRISMA) statement [14]. The review was registered with the International Prospective Register of Systematic Reviews (PROSPERO) in April 2020 (CRD42020135814). A systematic review was conducted as a meta-analysis was not feasible due to the considerable heterogeneity among study outcome measures.

### Search strategy

A systematic search was conducted with five scientific databases- Scopus, ERIC, MEDLINE (EBSCO), PsycINFO and Web of Science. Databases were searched from their inception to December 2021. Search terms were developed to capture all variations of the terminology related to before and after school programs as countries globally have varying names for the services. The initial search terms used were “out of school hours care” OR “outside school hours care” OR “out of school time program*” OR “after school care” OR “after school program*” OR “before school care” OR “before school program*” OR “breakfast club*” OR “after school club*” OR “wrap around care”; AND “healthy eating” OR food* OR nutrit* OR diet* OR “physical activity” OR movement OR exercise* OR sedentary OR sitting. These terms were tested for feasibility with Scopus before they were used in all databases. Nutrition related search terms were included as initially this review was also looking at healthy eating behaviours in OSHC services, however, only one study met the inclusion criteria. Consequently, we focused solely on PA and sedentary behaviours and excluded the healthy eating study.

Search records were extracted from the databases and imported into Endnote referencing software [15], where duplicate records were removed. Screening was conducted by multiple authors to reduce the risk of rejecting relevant articles [14]. Four independent reviewers screened the titles and abstracts of records against the eligibility criteria (AW, RC, LP, SR). Full text versions of studies meeting the criteria in initial screening were retrieved and assessed for final inclusion by three independent reviewers (AW, SR, LP), and their reference lists were manually searched to identify any additional relevant literature (AW). References found from the manual search also had the inclusion and exclusion criteria applied to determine relevance. Any discrepancies were resolved by discussion between reviewers, with an independent reviewer available for consultation if necessary (AO).

### Inclusion and exclusion criteria

Articles were included if they were: (1) written in the English language; (2) from a peer-reviewed academic journal; (3) contained data from a centre-based before and/or after school care service; (4) had a sample population of children with a mean age under 13 years (elementary/primary school age); (5) contained an objective measure of physical activity or sedentary behaviour; and (6) reported correlations or associations between the objective measure and other demographic, environmental, contextual or behavioural variables; and reported statistical significance (*p* value) of these correlations. Intervention studies reporting correlates at baseline would be eligible, however none did so they were excluded. Reviews, conference proceedings, dissertations and non-scholarly sources were also excluded from the review.

Consistent with other reviews of PA and sedentary behaviour correlates in children [8, 11], studies were required to have an objective measure of PA or sedentary behaviour. Physical activity is a complex behaviour, and research has demonstrated objective measures as more precise compared to subjective measures [16], particularly among children where issues with recall accuracy can arise [17]. Commonly used objective measurement tools reviewers were looking for were wearable monitors, indirect calorimetry and direct observation. Variables associated with the objective measure could be reported with both subjective or objective measures; in this way any contextual information captured by the subjective methods was included.

### Study risk of bias assessment

Individual study risk of bias was assessed by two independent authors (AW, SR) using the Office of Health Assessment and Translation (OHAT) Risk of Bias Rating Tool for Human and Animal Studies [18]. This tool assesses the risk of bias at the outcome level and rates cross-sectional studies using seven questions covering six types of bias: selection, confounding, attrition/exclusion, detection, selective reporting, and other. The risk of bias for each question was considered as definitely low, probably low, probably high and definitely high risk of bias. Initial review agreement between the two authors was low, at an agreement score of 47%. Most of the differences were between whether a criterion was ‘definitely low’ or ‘probably low’, and following a mutual discussion clarifying the definition of direct versus indirect evidence, these differences were resolved with an agreement score of 100%.

### Data extraction and synthesis

Data were extracted from each eligible study by one review author in a tabular format including: the country of study; the sample size; objective physical activity/sedentary behaviour data collection method/s; and any identified correlates (Table 1). A variety of techniques were used in the selected papers to report variables including univariate, bivariate and multilevel analyses. Similar to other reviews [10–12], for analyses focused on correlates where multiple analytic models were reported, findings from the final or fully adjusted models were extracted.

**Table 1:** Summary of included articles

Author/date	Location	Sample	Assessment and Outcome		Correlates of PA observed		Correlates of sedentary behaviour observed	
			**Outcome**	**Measurement tool**	**Variable**	**SEF Domain**	**Variable**	**SEF Domain**
Ajja et al., 2014 [19]	United States	20 ASPs; 1302 children (5–12 years old); 53.6% boys; 56.1% White	Minutes of MVPA (indoor/outdoor)	Actigraph accelerometer	% local pop. in poverty	Community	% local pop. in poverty	Community
			Minutes of sedentary behaviour	Actigraph accelerometer	Free play	Institutional	Age of child	Individual
			PA policy characteristics	HAPI-PA Scale	Ethnicity	Individual	BMI	Individual
					BMI	Individual	Ethnicity (non-white)	Individual
					Age of child	Individual	Size of used PA space	Institutional
					Size of used PA space	Institutional	Free play	Institutional
					Supportive PA policy characteristics	Institutional	Supportive PA policy characteristics	Institutional
Beets et al., 2012 [20]	United States	3 ASPs; 245 children (mean age 8.2 years old); 48% boys; 60% White	Minutes of MVPA	Actigraph accelerometer	Minutes in attendance	Individual		
			Steps	Walk4Life MVPA pedometer	Meeting 30 min MVPA guideline	Individual		
Beets et al., 2013 [21]	United States	18 ASPs; 1241 children (5–12 years old); 50% boys; 59% White	Minutes of MVPA	Actigraph accelerometer	PA evaluation (limited with nonvalid methods)	Institutional	Activities appealing to both genders	Institutional
			Minutes of sedentary behaviour	Actigraph accelerometer	PA policy (non-specific language)	Institutional	Scheduled PA time (< 25% schedule)	Institutional
			PA policy environment characteristics		Scheduled PA time (50% or more)	Institutional	Child feedback (formal collection)	Institutional
					Staff PA training (< 1 h)	Institutional	Child feedback (informal collection)	Institutional
					Staff PA training (1 to 4 h)	Institutional	Scheduled PA time (25–49% schedule)	Institutional
					Scheduled activities (limited)	Institutional	Scheduled PA time (50% or more)	Institutional
					Scheduled PA time (< 25% schedule)	Institutional	Staff PA training (< 1 h)	Institutional
					Child feedback (informal collection)	Institutional	PA curriculum (non-evidence based)	Institutional
					PA curriculum (non-evidence based)	Institutional	PA evaluation (limited with nonvalid methods)	Institutional
					Activities appealing to both genders	Institutional	PA policy (non-specific language)	Institutional
					Child feedback (formal collection)	Institutional	PA training delivered by noncertified personnel	Institutional
					Scheduled activities (diverse)	Institutional	Scheduled activities (diverse)	Institutional
					PA training delivered by noncertified personnel	Institutional	Scheduled activities (limited)	Institutional
					Scheduled PA time (25–49% schedule)	Institutional	Staff PA training (1 to 4 h)	Institutional
Beets et al., 2015 [22]	United States	19 ASPs; 812 children (6–12 years old); 53% boys; 61% White non-Hispanic	Minutes of total PA	Actigraph accelerometer	BMI	Individual		
			Minutes of MVPA	Actigraph accelerometer	School-based ASP	Institutional		
			Meeting PA policy benchmarks		Ethnicity (non-white)	Individual		
					Age of child	Individual		
					Attended faith-based ASP	Institutional		
					Sex	Individual		
Beets et al., 2016 [23]	United States	20 ASPs; 1408 children (5–12 years old); 56% boys	Minutes of MVPA	Actigraph accelerometer	% PA opportunities dedicated for free play (45–74%)	Institutional	% PA opportunities dedicated for free play (45–74%)	Institutional
			Minutes of sedentary behaviour	Actigraph accelerometer	Scheduled PA time (110–160 min)	Institutional	% PA opportunities dedicated for free play (75–100%)	Institutional
			Program characteristics	Review of schedules; direct observation	Scheduled PA time (90–105 min)	Institutional	Annual organisation revenue (approx $2–4 million)	Institutional
					Sedentary option during PA time (60 min)	Institutional	Annual organisation revenue (approx $5–20 million)	Institutional
					Sedentary option during PA time (75–120 min)	Institutional	Attending YMCA ASP	Institutional
					Annual organisation revenue (approx $2–4 million)	Institutional	Scheduled PA time (110–160 min)	Institutional
					Annual organisation revenue (approx $5–20 million)	Institutional	Scheduled PA time (90–105 min)	Institutional
					Attending YMCA ASP	Institutional	Sedentary option during PA time (60 min)	Institutional
					Outdoor PA space (approx 231 k-281 k ft^2^)	Institutional	Sedentary option during PA time (75–120 min)	Institutional
					Outdoor PA space (approx 85 k-200 k ft^2^)	Institutional	Indoor PA space (4461–6009 ft^2^)	Institutional
					% PA opportunities dedicated for free play (75–100%)	Institutional	Indoor PA space (7344–15,056 ft^2^)	Institutional
					Indoor PA space (4461–6009 ft^2^)	Institutional	Outdoor PA space (approx 231 k-281 k ft^2^)	Institutional
					Indoor PA space (7344–15,056 ft^2^)	Institutional	Outdoor PA space (approx 85 k-200 k ft^2^)	Institutional
					Staff PA training (1 or more hours)	Institutional	Staff PA training (1 or more hours)	Institutional
Brazendale et al., 2015 [24]	United States	20 ASPs; 1248 children (5–12 years old); 53% boys	Scheduled PA time	Schedule review	Scheduled PA time (< 60 min)	Institutional		
			Minutes of MVPA	Actigraph accelerometer	Scheduled PA time (> 60 min)	Institutional		
					Scheduled PA time (≈ 45 min)	Institutional		
					Scheduled PA time (≤ 30 min)	Institutional		
					Scheduled PA time (≥ 105 min)	Institutional		
					Scheduled PA time (60 min)	Institutional		
					Scheduled PA time (75 min)	Institutional		
Burrows et al., 2014 [25]	Canada	2 ASPs; 40 children (6–10 years old); 42% boys	FMS proficiency	Test of Gross Motor Development 2	Attended sport-based ASP	Institutional		
					Low-organised game ASP	Institutional		
Crowe et al., 2021 [26]	Australia	89 ASPs; 4408 children (5–12 years old); 42% boys	Child physical activity	Actigraph accelerometer	Sex	Individual	Sex	Individual
			Physical activity policies	HAAND	Grade	Individual	Grade	Individual
			Physical activity promotion practices	SOSPAN	PA policy	Institutional		
			Type and structure of physical activity	SOSPAN	Staff PA training	Institutional		
			Available PA space	Craftright measuring wheel	PA promotion material	Institutional		
					Screen time availability	Institutional		
					Handheld device availability	Institutional		
					Children input in daily programming	Institutional		
					Scheduled free play	Institutional		
					Organised PA	Institutional		
					Children engaged in PA	Individual		
					Children stand and wait during PA games	Interpersonal		
					Elimination games	Institutional		
					Staff engaged in PA	Interpersonal		
Domazet et al., 2015 [27]	Denmark	10 ASPs; 475 children (5–11 years old); 41% boys	MVPA minutes	Actigraph accelerometer	Attended regular ASP	Institutional	Attended non sport-based ASP	Institutional
			Cardiovascular fitness		Attended sport-based ASP	Institutional	Attended sport-based ASP	Institutional
Huberty et al., 2013 [28]	United States	12 ASPs; 888 children (mean age 8.7 years old); 44% boys; 77% White non-Hispanic	Staff behaviour	SOPLAY	Minutes of scheduled PA	Institutional		
			Scans observed sedentary	SOPLAY	Organised PA	Institutional		
			Scans observed walking	SOPLAY	PA equipment available	Institutional		
			Scans observed vigorous	SOPLAY	Staff engaged in PA	Interpersonal		
					Staff off task during PA	Interpersonal		
					Staff other duties during PA	Interpersonal		
					Staff promoting PA	Interpersonal		
					Total number of boys/girls	Institutional		
Kuritz et al., 2020 [29]	Germany	ASPs; 198 children	% of time in sedentary, LPA & MVPA	Actigraph accelerometer	Minutes in attendance	Individual	Sex	Individual
			Socio-demographic data	Motorik-Modul activity questionnaire	Sex	Individual	Grade (year 1 only)	Individual
					Age of child	Individual	Minutes in attendance	Individual
Londal et al., 2020 [30]	Norway	14 ASPs; 42 children (Grade 1); 52% boys	Time in sedentary behaviour, total PA and MVPA	Actigraph accelerometer	Outdoor PA (comparison indoor)	Institutional	Sex	Individual
			PA periods	Observation form	Sex	Individual		
Maher et al., 2019 [31]	Australia	23 ASPs; 1068 children	Service contexual information and policies	HAAND	Number of active play zones available	Institutional	% of session in MVPA	Institutional
			Sedentary, LPA, MVPA	SOPLAY	Outdoor play duration	Institutional	Availability of screen before 5 pm	Institutional
			Staff PA and nutrition promotion behaviours	SOSPAN	Screen availability	Institutional	Screen availability	Institutional
					Total number of screen devices	Institutional	Service size	Institutional
					Availability of screens before 5 pm	Institutional	Number of active play zones available	Institutional
					Service size	Institutional	Outdoor play duration	Institutional
					Staff promoting PA	Interpersonal	Staff promoting PA	Interpersonal
					Staff witholding PA	Interpersonal	Staff witholding PA	Interpersonal
							Total number of screen devices	Institutional
Riiser et al., 2019 [32]	Norway	14 ASPs; 426 children (Grade 1); 52% boys	Physical activity	Actigraph accelerometer	BMI	Individual	BMI	Individual
			Child biometrics		Sex	Individual	Sex	Individual
Rosenkranz et al., 2011 [33]	United States	7 ASPs 230 children (grade 3 and 4)	Physical activity	Actigraph accelerometer	Child self-efficacy	Individual		
					PA enjoyment	Individual		
					Sex	Individual		
					BMI	Individual		
					Parent PA social support	Interpersonal		
					Child socioeconomic status	Individual		
					Ethnicity (non-white)	Individual		
Trost et al., 2008 [34]	United States	7 ASPs; 147 children (mean age 10.1 years old); 54% male	Physical activity	Actigraph accelerometer	BMI	Individual	BMI	Individual
			Height and weight	Anthropometric measures	Sex	Individual	Sex	Individual
					Indoor free play (comparison academic time)	Institutional		
					Indoor free play (comparison indoor organised PA)	Institutional		
					Indoor free play (comparison outdoor free play)	Institutional		
					Indoor free play (comparison outdoor organised PA)	Institutional		
					Indoor free play (comparison snack time)	Institutional		
					Indoor organised PA (comparison academic time)	Institutional		
					Indoor organised PA (comparison outdoor free play)	Institutional		
					Indoor organised PA (comparison outdoor organised PA)	Institutional		
					Indoor organised PA (comparison snack time)	Institutional		
					Outdoor free play (comparison academic time)	Institutional		
					Outdoor free play (comparison outdoor organised PA)	Institutional		
					Outdoor free play (comparison snack time)	Institutional		
					Outdoor organised PA (comparison academic time)	Institutional		
					Outdoor organised PA (comparison snack time)	Institutional		
					Snack time (comparison academic time)	Institutional		
Weaver et al., 2014 [35]	United States	4 ASPs; Undisclosed	Staff PA and nutrition promotion behaviours	SOSPAN	Children stand and wait during PA games	Institutional	Scheduled enrichment	Institutional
			Scans observed sedentary	SOPLAY	Elimination PA games	Institutional	Children stand and wait during PA games	Institutional
			Scans observed walking	SOPLAY	Idle time	Institutional	Elimination PA games	Institutional
			Scans observed vigorous	SOPLAY	Scheduled academics	Institutional	Scheduled snack	Institutional
					Scheduled activity rotation	Institutional	Idle time	Institutional
					Scheduled bathroom	Institutional	Scheduled academics	Institutional
					Scheduled enrichment	Institutional	Scheduled activity rotation	Institutional
					Scheduled snack	Institutional	Scheduled bathroom	Institutional
					Staff discipline children	Interpersonal	Staff discipline children	Interpersonal
					Staff discouraging PA	Interpersonal	Staff discouraging PA	Interpersonal
					Staff engaged in PA	Interpersonal	Staff engaged in PA	Interpersonal
					Staff giving instructions during PA	Interpersonal	Staff giving instructions during PA	Interpersonal
					Staff leading PA	Interpersonal	Staff leading PA	Interpersonal
					Staff off task during PA	Interpersonal	Staff off task during PA	Interpersonal
					Staff other duties during PA	Interpersonal	Staff other task during PA	Interpersonal
					Staff promoting PA	Interpersonal	Staff promoting PA	Interpersonal
					Staff witholding PA	Interpersonal	Staff witholding PA	Interpersonal
Zarrett et al., 2015 [36]	United States	7 ASPs; Undisclosed (7–12 years old); 56% male	PA levels in METS	SOPLAY	Average temperature	Community		
			Social and environment context of PA	SOPLAY	Children appear engaged	Individual		
			Social-motivational climate of ASPs	MCOT-PA	Clear PA rules	Institutional		
					Free play	Institutional		
					Organised PA	Institutional		
					PA activity includes most children	Institutional		
					PA equipment available	Institutional		
					Staff encouraging out-of-program PA	Interpersonal		
					Staff engaged in PA	Interpersonal		
					Staff leading PA	Interpersonal		
					Staff promoting PA	Interpersonal		
					Staff supervision	Interpersonal		
					Staff supervising PA	Interpersonal		
					Usable environment	Institutional		
					Youth interacting positively with one another	Interpersonal		

*Abbreviations: ASPs* after school programs, *BMI* body mass index, *HAAND* Healthy Afterschool Activity and Nutrition Documentation, *HAPI-PA* Healthy Afterschool Program Index-Physical Activity scale, *LPA* light physical activity, *MCOT-PA* Motivational Climate Observation Tool for Physical Activity, *MPA* moderate physical activity, *MVPA* moderate to vigorous physical activity, *PA* physical activity, *SEF* social ecological framework, *SOPLAY* System for Observing Play and Leisure in Youth, *SOSPAN* System for Observing Staff Promotion of Physical Activity and Nutrition

The correlates were categorised by one review author (AW) into their associated social ecological framework domain: individual, interpersonal, institutional, community and public policy [13]. A second review author (JN) reviewed the categorisation and any discrepancies were discussed and consensus reached. Consistent with other reviews of PA and sedentary behaviour in children [10–12] the social ecological framework was used to allow for the investigation of multidimensional factors that influence PA and sedentary behaviour; and provide an organised approach to inform future interventions in the OSHC setting [13]. In the context of this review, the institutional domain refers to correlates at the individual OSHC service provider level, whereas the community domain refers to correlates that are external to the service and from the wider society.

Correlates were summarised to determine shared associations (see Additional files 1 and 2). Correlates which reported a statistically significant (*p* < 0.05) association with a PA or sedentary behaviour outcome measure were coded as + or – depending on the association (Column 4, Additional files 1 and 2). Those reporting no significant association were recorded in Column 5. The number of times a correlate was associated with an outcome variable was tallied against the total number of times the association was observed (including studies with no significant association). The tally was converted into a percentage (Column 7, Additional files 1 and 2) and analysed using a summary code to represent the association (Table 2). This was similar to a previously published extraction and synthesis process [11] and method of coding [10, 12]. This summary code for the overall association was then recorded (Column 8, Additional files 1 and 2) and used for discussion of the results.

**Table 2:** Rules for classifying variables regarding strength of association

Outcome measures supporting association (%)	Summary code	Explanation of code
0–33	0	Non-significant association
34–59	?	Inconclusive association
60–100	+	Positive association
60–100	-	Negative association

Note: When a correlation was observed in three or more studies, it was coded as: 00 (non-significant association for three or more studies); ?? (inconclusive for three or more studies); +  + (positive association for three or more studies); – (negative association for three or more studies). This assists visually with correlations that were more widely studied

### Reporting of outcome findings

The reporting of outcome findings in the results is presented using the summary coding for each correlate (Column 7, Additional files 1 and 2). Accordingly, (n/N) refers to the number of significant associations found with outcome measures / total number of associations studied (for that particular correlate). The literature cited refers to the studies which reported the summary code relationship for that correlate (i.e. no association, indeterminate association, positive association, negative association).

## Results

**Fig. 1:**
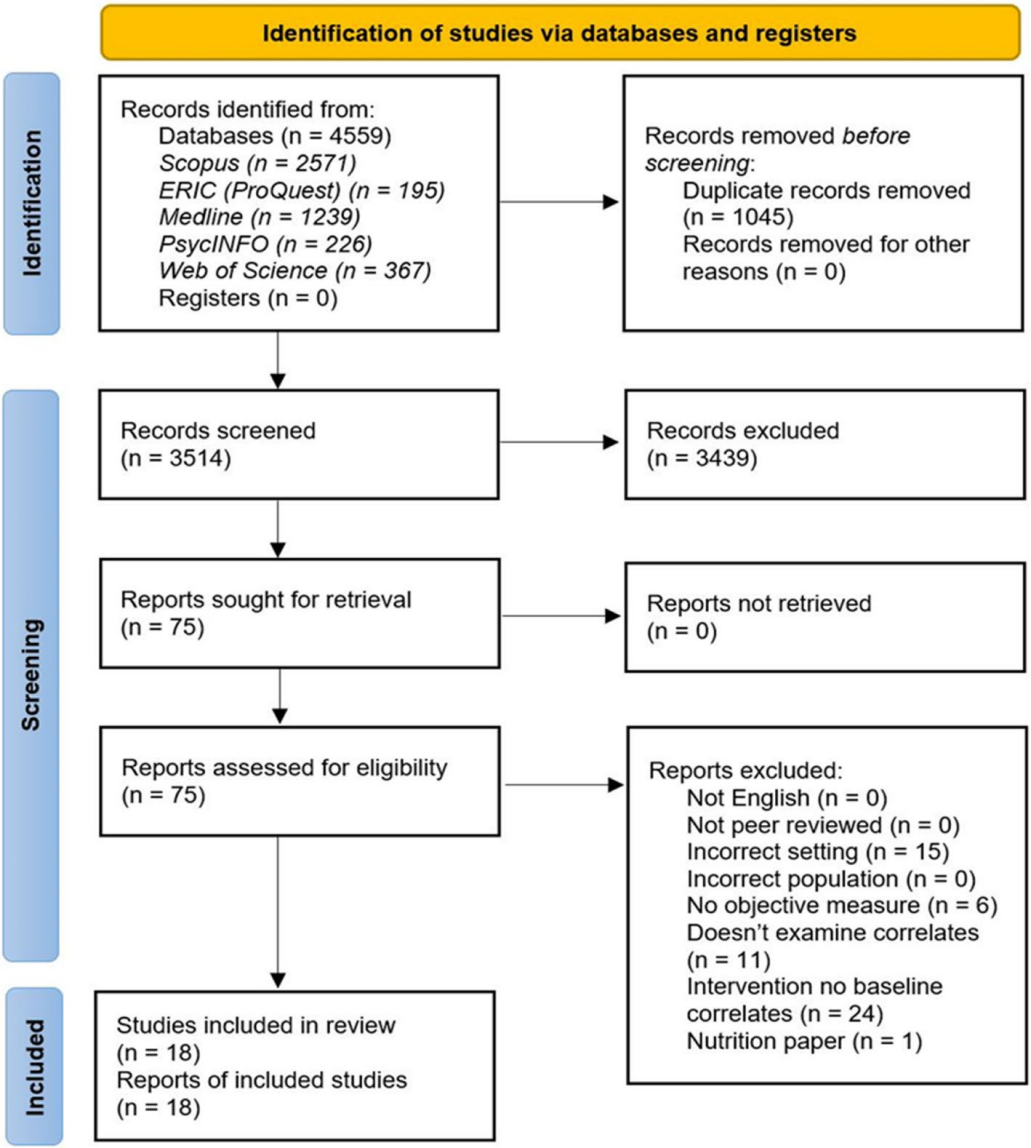
PRISMA flow diagram for the search results and inclusions process for identification of articles

A total of 4559 papers were retrieved with 3514 remaining after duplicates were removed (Fig. 1). Following the title and abstract screening, 75 studies were retrieved for full-text review. Of these, 18 studies met the inclusion criteria and were included in this review (Table 1). Publication years of included studies ranged from 2008 – 2021, with all but two studies [33, 34] published in the last 10 years. Most studies (61%) were conducted in the United States (*n* = 11) [19–24, 28, 33–36]. The remainder were from Australia (*n* = 2) [26, 31], Norway (*n* = 2) [30, 32], Canada (*n* = 1) [25], Denmark (*n* = 1) [27] and Germany (*n* = 1) [29]. All studies were conducted in after school programs (*n* = 18) with no studies in before school care settings. As the decision to remove healthy eating from this review was made after the screening was completed, the numbers indicated in the PRISMA flow diagram (Fig. 1) include studies which met the healthy eating search terms indicated in the search strategy of the methods section.

### Risk of bias in studies

**Fig. 2:**
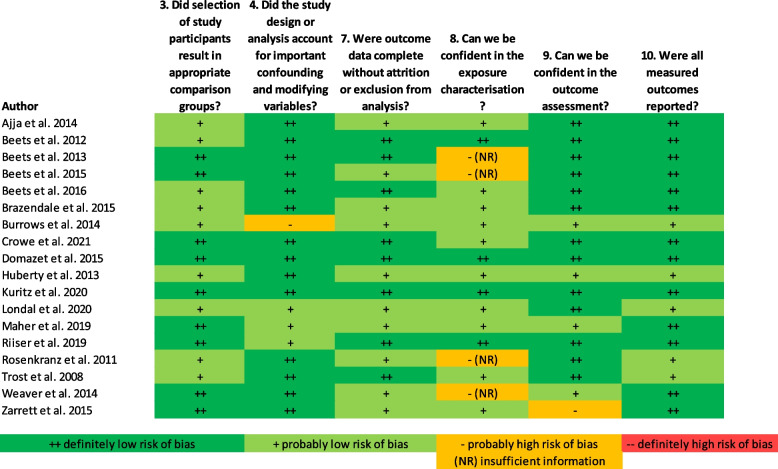
Risk of bias in individual studies, assessed using the OHAT Risk of Bias Rating Tool [18]

All included studies had an overall ‘definitely low’ or ‘probably low’ risk of bias (Fig. 2). No studies had any criteria which were ‘definitely high’ risk of bias, and only six studies had one ‘probably high’ risk of bias criterion [21, 22, 25, 33, 35, 36]. The most common ‘probably high’ risk of bias was related to the exposure characterisation, with four studies using invalidated methods to measure the exposure(s) [21, 22, 33, 35]. Due to these exposure measures being indirect, the tool called for ‘(NR)’ to be recorded which indicates there was insufficient information to assess risk.

### Summarising the studies

PA and sedentary behaviour were assessed using a range of measurement methods. Thirteen studies used accelerometers [19–24, 26, 27, 29, 30, 32–34], one study used pedometers [20], and six studies used direct observations via three tools—System for Observing Play and Leisure Activity in Youth (SOPLAY) (*n* = 4) [28, 31, 35, 36], Test of Gross Motor Development 2 (*n* = 1) [25], and one other unnamed observation tool (*n* = 1) [30]. Correlational information on the PA and sedentary behaviour environments was collected from policy reviews using five tools: System for Observing Staff Promotion of Activity and Nutrition (SOSPAN) (*n* = 3) [26, 31, 35], Healthy Afterschool Program Index-Physical Activity (HAPI-PA) scale (*n* = 1) [19], Healthy Afterschool Activity and Nutrition Documentation (HAAND) (*n* = 2) [26, 31], a PA policy environment framework (*n* = 1) [21] and policy benchmarks (*n* = 1) [22]. Other correlational information was collected from a review of the service schedule (*n* = 2) [23, 24], an unnamed psychosocial questionnaire (*n* = 1) [33], Motivational Climate Observation Tool for Physical Activity (MCOT-PA) (*n* = 1) [36], and administrative records (*n* = 1) [33].

A total of 116 correlates of PA were identified (Additional file 1), of which 10 were classified as individual, 14 as interpersonal, 90 as institutional and two as community variables. There were 64 correlates of sedentary behaviour identified (Additional file 2), of which six were classified as individual variables, nine as interpersonal, 48 as institutional and one as a community variable. Identified associations reflected the relationship between the correlate and PA or sedentary behaviour outcome stated in Column 3 (Additional files 1 and 2).

### Summarising the outcome findings

#### Individual variables

Ten individual level correlates relating to PA were identified (Additional file 1). The most frequently observed individual correlates were sex and BMI. Seven studies [22, 26, 29, 30, 32–34] reported 30 associations between sex and varying PA outcomes, six of which found 20 significantly positive associations (*n* = 20/30) [22, 26, 29, 32–34]. This indicates a positive association between sex and PA, with males more physically active than females. Five studies [19, 22, 32–34] reported 26 associations with BMI, of which only four studies found five which were significantly negative (*n* = 5/26) [19, 22, 32, 34] indicating an overall null association. An inconclusive association was also found between PA and age, with three studies reporting eight significant negative associations out of 16 total (*n* = 8/16) [19, 22, 26].

Six individual level correlates relating to sedentary behaviour were also identified (Additional file 2), with the most frequently observed being sex, BMI and age. Five studies reported eight associations between sex and sedentary behaviour, six of which were significantly negative indicating an overall negative association and showing that females were more sedentary than males (*n* = 6/8) [26, 29, 30, 32]. An overall inconclusive association was found between BMI and sedentary behaviour, with three studies reporting seven non-significant associations (*n* = 0/7) [19, 32, 34]. Two studies revealed an overall positive association between age and sedentary behaviour (*n* = 4/6) [19, 26], finding older children more sedentary.

#### Interpersonal variables

There were 14 interpersonal level correlates relating to PA (Additional file 1). The most commonly reported was staff verbally promoting PA with four studies [28, 31, 35, 36] reporting 13 correlates, of which only two were significant (*n* = 2/13) [28, 36] revealing an overall non-significant association. Staff being engaged in PA was found to have an overall positive association with PA outcomes, as two studies reported seven significantly positive associations (*n* = 7/10) [35, 36]. One study also found that staff supervision of PA (*n* = 3/3) and children interacting positively with each other (*n* = 2/3) had positive associations on PA [36].

Nine interpersonal correlates relating to sedentary behaviour were reported (Additional file 2). One study found positive associations between staff disciplining children during PA (*n* = 2/2), staff discouraging PA (*n* = 2/2) and staff giving instructions during PA (*n* = 2/2) with sedentary behaviour outcomes [35]. The same study also reported a negative association between staff engaged in PA and sedentary behaviour (*n* = 2/2) [35].

#### Institutional variables

Ninety institutional correlates of PA were identified (Additional file 1). The most frequently studied related to the associations between activity structure (organised PA and free play) and PA outcomes. The results were inconclusive. Two studies reported seven associations between free play and PA, with only two being significant (*n* = 2/7) [19, 36] revealing an overall non-significant association. Two studies [28, 36] also reported eight associations between organised PA and PA outcomes, and only one was reported as significant (*n* = 1/8) [36] indicating another non-significant association. PA games with an elimination component were found to be associated with reduced PA levels, as two studies reported five negative associations between elimination PA games and PA (*n* = 5/5) [26, 35]. PA equipment availability is associated with increased PA, with two studies reporting four positive associations (*n* = 4/5) [28, 36]. Scheduling PA time in OSHC was also found associated with increased PA, with one study finding a positive association between scheduled PA time of 60 and 75 min with PA (*n* = 2/2) [24], another study finding a positive association with scheduled PA time between 90–105 min and PA (*n* = 4/4) [23], and one more finding a positive association with scheduling 50% or more of the session for PA and PA outcomes (*n* = 2/2) [21]. Another study also found that scheduling 30 min or more of free play (*n* = 1/1) [26] and organised PA (*n* = 1/1) [26] was found positively associated with PA.

Forty-eight institutional correlates of sedentary behaviour were identified (Additional file 2). One study found two positive associations between elimination-based PA games and sedentary behaviour (*n* = 2/2) [35], however conversely found two positive associations between children standing and waiting during PA games and sedentary behaviour (*n* = 2/2) [35]. Scheduling 50% or more of the OSHC session for PA time was found to have an overall negative association on sedentary behaviour, with one study finding two negative associations (*n* = 2/2) [21]. Screen time was also found to be associated with increased sedentary behaviour, with a study finding that screen availability and the total number of screen devices in a service both increase the percentage of the session children spend in screen time (*n* = 1/1) [31].

#### Community variables

There were two community level correlates of PA identified (Additional file 1). One study found a non-significant association between percentages of the local population living in poverty and PA (*n* = 0/4) [19] and another found a non-significant association between the average temperature and PA (*n* = 0/3) [36].

One community correlate of sedentary behaviour was identified (Additional file 2). This consisted of one study reporting four associations between percentage of the local population in poverty and sedentary behaviour, of which only one was significantly positive resulting in an overall non-significant association (*n* = 1/4) [19].

#### Public policy variables

No extracted correlates were categorised into the public policy domain.

## Discussion

To the best of our knowledge, this is the first systematic review that reports the correlates of objectively measured physical activity and sedentary behaviour in OSHC services. This review demonstrated the varying social ecological domains which were associated with physical activity and sedentary behaviour, similar to other reviews [11, 12]. Physical activity correlates were most frequently reported, however, sedentary behaviour was often addressed in conjunction. The majority of the extracted correlates were categorised into the institutional domain, followed by the interpersonal, individual and community domains respectively. This demonstrates the priority areas interventions within the OSHC setting should target.

The individual domain demonstrated an association that males engage in more PA and are less sedentary than females, which is consistent with reviews of children in other settings [10, 11]. This highlights a need for OSHC services to better engage female children in physical activity, possibly through programming activities which appeal to both sexes. This idea is consistent with a correlate found in the institutional domain, which found programming activities which appeal to both sexes is associated with increased PA among females [20]. This relationship between correlates is an example of the interactions which exist between the social ecological domains, and how looking at PA and sedentary behaviour in the OSHC setting through this framework offers an insightful approach for future interventions.

The interpersonal domain revealed correlates of PA and sedentary behaviour which was anticipated. Staff engaging in and supervising PA was associated with increased physical activity levels [35, 36], and staff discouraging PA and disciplining children was significantly associated with increased sedentary behaviour [35]. The discouragement of PA and discipline of children being associated with more time spent sedentary is an implied relationship, which makes it concerning that staff are actively engaging in this behaviour. Staff training and service policies to promote staff engaging in and supervising PA and educating staff not to discourage children while they are physically active should be an approach for all OSHC services.

In this review, the institutional domain provided most insight into the correlates of PA and sedentary behaviour in OSHC. PA games which involve elimination were associated with reduced PA and increased sedentary behaviour [26, 35], something commonly seen and a game element studies recommend against using [37]. Increased PA equipment availability was also associated with higher PA levels [28, 36]. While availability of equipment is dependent upon finances, services should explore their options around acquiring and providing additional PA equipment through fundraising and other means. OSHC services also need to prioritise scheduling dedicated PA time into their daily programming, as several studies found associations between higher levels of scheduled PA and reduced sedentary behaviour and increased PA levels [21, 23, 24].

Findings around activity structure through the impact of free play and organised PA were mixed, with several studies exploring these factors and finding no significant associations or conflicting results [19, 28, 34, 36]. Further research should be conducted to determine more definitively the association between activity structure and PA and sedentary behaviour in OSHC services. One institutional correlate which was unexpected was children standing and waiting during PA games being associated with higher levels of PA and lower levels of sedentary behaviour [35]. It is, however, important to note that this was only found in one study and was attributed to the complex nature of the OSHC program setting with many events happening simultaneously possibly causing this contradictory relationship [35].

All studies were based in the after school care setting, with no studies included from the before school care setting. This was not unexpected, as preliminary literature searches found most of the research conducted in the OSHC setting was from the United States, and searches for before school care in the United States revealed very little information, suggesting that it is not a prominent setting in that country. Before school care is, however, common in countries such as Australia where there are 4258 registered services who offer this care [38], and New Zealand where 8% of children 6–12 years attend before school care [39]. This reveals another gap in the literature and a need for more studies in OSHC based outside of the United States.

It is important to note that this review initially included correlates of healthy eating in the OSHC setting, though was modified when only one study met the inclusion criteria [40]. While there were a few studies on the food environment of OSHC identified, an inclusion criteria for this review was an objective measure, and most of the studies either did not look at the consumption of food or the measures were subjective in nature. While the screening criteria of this study may have been too stringent to explore the healthy eating environments of the OSHC setting, it does reveal a gap in the literature of a lack of objective healthy eating studies in OSHC services.

### Limitations

The results of this review should be considered in light of a number of limitations, including: 1) there were only a small number of studies for most variables; 2) most of the studies were from the United States and may limit the generalisability of the results; 3) none of the included studies observed the before school care setting, meaning the findings may not be representative of that sector; 4) the studies reviewed varied in sample size, outcome measures, and methodologies (although all used an objective measure of PA or sedentary behaviour), which may impact the heterogeneity of the estimates and likelihood of biases in conclusions made; 5) only studies which used an objective measure of PA or sedentary behaviour were included in this review, therefore findings from studies using subjective measures were not accounted for and could vary some of the conclusions made in this study; 6) there was only one author responsible for extracting data from included studies and, though this process was undertaken with extreme diligence, there is potential for error.

## Conclusions

This review is important as a large number of children aged 5–13 years attend before and/or after school care services [4], and the sector has been identified as having the potential to positively influence the physical activity, sedentary behaviour and heathy eating of children [5, 6, 41]. This review provides an understanding of the diverse range of influences in participation in physical activity and sedentary behaviour among children while attending OSHC services. It reinforces that females are often less physically active and more sedentary than males in these environments, with service providers and staff needing to explore ways to further engage female children in PA. Service providers also need to monitor staff behaviours around PA through means such as training and policy, as it has the potential to both positively and negatively influence how active children are. They should also look towards removing elimination elements from their PA games, try to schedule more time for PA and also provide more PA equipment for children to use. Health researchers need to look further into how activity structure impacts on child PA, as current studies report mixed findings. This review addresses a knowledge gap and will contribute to future research in both the OSHC setting and childhood overweight and obesity prevention.

## Supplementary Information

**Additional file 1.** Summary of reported physical activity correlates.

**Additional file 2.** Summary of reported sedentary behaviour correlates.

**Additional file 3. **Search strategy.

## Data Availability

The datasets supporting the conclusions of this article are included within the article (and its additional files).

## Abbreviations

BMI: Body mass index
HAAND: Healthy Afterschool Activity and Nutrition Document
HAPI-PA: Healthy Afterschool Program Index-Physical Activity
MCOT-PA: Motivational Climate Observation Tool for Physical Activity
MVPA: Moderate-to-vigorous physical activity
OECD: Organisation for Economic Co-operation and Development
OHAT: Office of Health Assessment and Translation
OSHC: Out of school hours care
PA: Physical activity
PRISMA: Preferred Reporting Items for Systematic Reviews and Meta-analysis
PROSPERO: International Prospective Register of Systematic Reviews
SOPLAY: System for Observing Play and Leisure Activity in Youth
SOSPAN: System for Observing Staff Promotion of Physical Activity and Nutrition
