# Supplementary material for: Correlates of physical activity and sedentary behaviour in children attending before and after school care: a systematic review
Source: BMC Public Health. 2022 Dec 16;22:2364. doi: 10.1186/s12889-022-14675-8 (PMC9758811; doi:10.1186/s12889-022-14675-8)
Supplement: Supplementary file 3 — Additional file 3. Search strategy. [file 12889_2022_14675_MOESM3_ESM.docx]

**Additional file 3-** Search strategy

**Article title** - Correlates of physical activity and sedentary behaviour in children attending before and after school care: A systematic review

**Search Terms**

“Out of school hours care” OR “outside school hours care” OR “out of school time program*” OR “after school care” OR “after school program*” OR “before school care” OR “before school program*” OR “breakfast club*” OR “after school club*” OR “wrap around care”

AND

“Healthy eating” OR food* OR nutrit* OR diet* OR “Physical activity” OR movement OR exercise* OR sedentary OR sitting

**Databases (searched from inception to December 2021)**

| **Databases** |
| --- |
| Scopus |
| ERIC |
| MEDLINE (EBSCO) |
| PsycINFO |
| Web of Science |

**Inclusion Criteria**

1. Written in the English language.
2. From a peer-reviewed academic journal.
3. Contained data from a centre-based before and/or after school care service.
4. Had a sample population of children with a mean age under 13 years (elementary/primary school age).
5. Contained an objective measure of physical activity or sedentary behaviour.
6. Reported correlations or association between the objective measure and other demographic, environmental, contextual or behavioural variables; and reported statistical significance (p value) of these correlations.
7. If study design was an intervention, then correlates were reported at baseline.
